# Modeling small cell lung cancer (SCLC) biology through deterministic and stochastic mathematical models

**DOI:** 10.18632/oncotarget.25360

**Published:** 2018-05-25

**Authors:** Ravi Salgia, Isa Mambetsariev, Blake Hewelt, Srisairam Achuthan, Haiqing Li, Valeriy Poroyko, Yingyu Wang, Martin Sattler

**Affiliations:** ^1^ City of Hope, Department of Medical Oncology and Therapeutics Research, Duarte 91010, CA, USA; ^2^ City of Hope, Center for Informatics, Duarte 91010, CA, USA; ^3^ Dana-Farber Cancer Institute, Department of Medical Oncology, Boston 02215, MA, USA; ^4^ Harvard Medical School, Department of Medicine, Boston 02115, MA, USA

**Keywords:** small cell lung cancer, computational modeling, discrete model, continuous model, systems biology

## Abstract

Mathematical cancer models are immensely powerful tools that are based in part on the fractal nature of biological structures, such as the geometry of the lung. Cancers of the lung provide an opportune model to develop and apply algorithms that capture changes and disease phenotypes. We reviewed mathematical models that have been developed for biological sciences and applied them in the context of small cell lung cancer (SCLC) growth, mutational heterogeneity, and mechanisms of metastasis. The ultimate goal is to develop the stochastic and deterministic nature of this disease, to link this comprehensive set of tools back to its fractalness and to provide a platform for accurate biomarker development. These techniques may be particularly useful in the context of drug development research, such as combination with existing omics approaches. The integration of these tools will be important to further understand the biology of SCLC and ultimately develop novel therapeutics.

## KEY POINTS

Novel therapies are urgently needed for patients with small cell lung cancer (SCLC)

The use of mathematical models in medicine is becoming progressively more robust due to the exponentially increasing processing power of new hardware and software

Computational models thus far have mostly focused on comprehensive cancer biology models rather than individualized disease models

Mathematical modeling integrated with computational modeling can be used to simulate tumor growth, mutational heterogeneity, cellular automata, and mechanisms of metastasis

Mathematical oncogenic models can provide insight into biological mechanisms of action, identification of biological markers, quantification of image analysis and understanding of chaotic cell dynamics, especially in immune function

## MAIN

**Figure 1:**
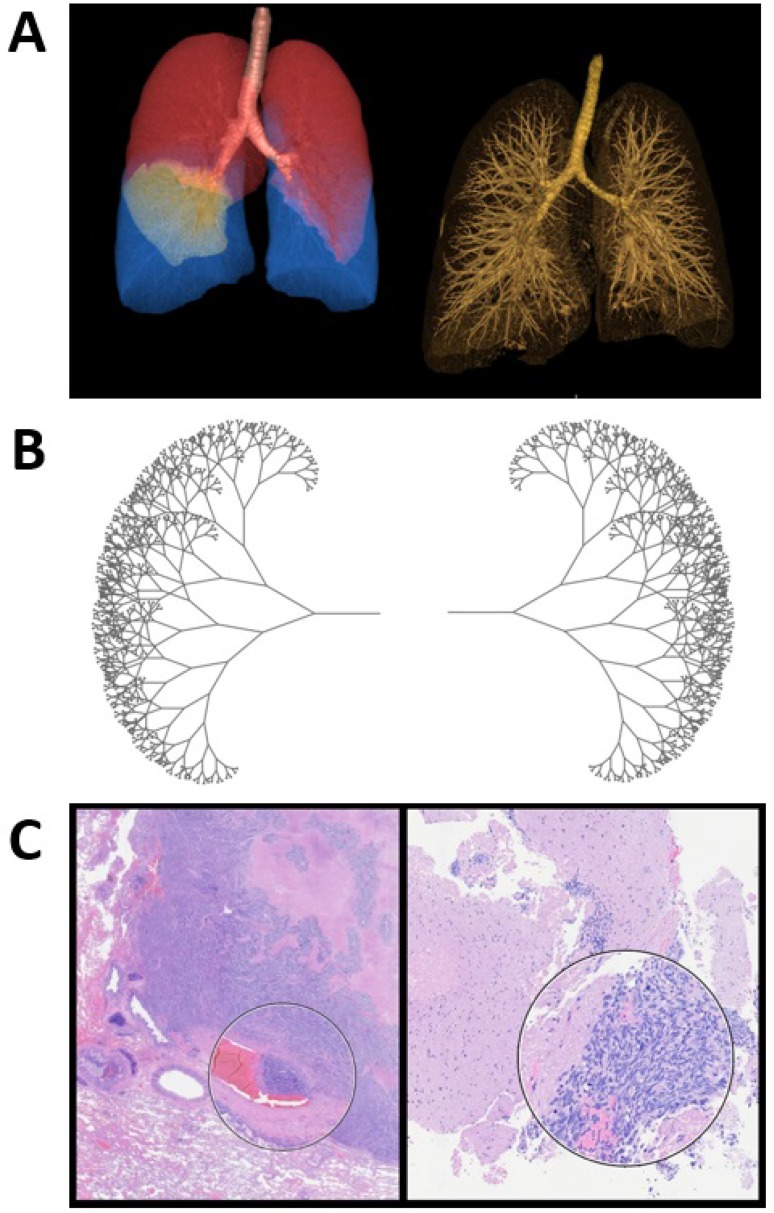
Fractal geometry in SCLC (**A**) 3D visualization of SCLC patient lungs and their fractal properties. (**B**) Fractal tree generated using Swift 3.1 programming language. (**C**) Histology of SCLC showing the fractal geometry of tumor tissue. Hematoxylin and eosin stained SCLC specimen. SCLC tends to travel in clusters and the encircled areas are examples of this clustered tumor property. Specifically, a cluster infiltrating into a blood vessel is shown on the left.

Self-similar, repeating patterns, also known as fractals, can describe the universe and mimic nature's highly complex structures. Nature often presents itself in fragments of patterns that are similar, yet not identical. The conservation of structural patterns in nature is manifested across organisms from different realms. One prominent example is the respiratory analogy between trees and lungs, where conservation of both fractal design (self-similarity) and function is elegantly manifested (Figure 1). The central tree trunk/trachea divides into wider branches/bronchi, which bifurcate into increasingly smaller branches/bronchioles, and eventually conclude in leaves/alveoli. The synchrony in morphology is also translated into function, where gas exchange occurs at the same terminal end, leaves or alveoli. Maintenance of this stable, yet complex natural order controls biological equilibrium and thus life. Quantifying changes in chaotic patterns could provide a valuable diagnostic tool to distinguish at an early stage between healthy and abnormal tissue, paving the way for more effective and personalized cancer treatments.

Our goal is to develop an understanding of the mathematical basis of cancer in the context of small cell lung cancer (SCLC) growth, mutational heterogeneity, and mechanisms of metastasis. The results can serve as a biomimetic framework for organizing and applying the wealth of available genomic and clinical data. We aim to communicate this mathematical understanding to clinicians and tumor biologists in the hope of generating precise treatment maps and therapies with improved outcomes. It is hypothesized that cancer is switched on by a chaotic imbalance through stochastic processes of mutation generation and genetic drift, which are inherently random. However, further malignant development seems to recover this balance, albeit to one far from normal, in favor of a deterministic process through clonal selection. Deterministic models include ordinary and partial differential equations that describe tumor kinetics and stroma, whereas stochastic models include cancer initiation and progression such as cellular automata and group theory. These tools may provide a focal point for integrated systems approaches that are aimed at the development of new cancer therapeutics.

## SMALL CELL LUNG CANCER (SCLC) AND ITS CLINICAL STATUS

SCLC is thought to originate from neuroendocrine cells that normally reside in the lung epithelium [1], still retaining some of the neuroendocrine cell markers, including CD56 [2, 3], chromagranin, and synaptophysin [1] or the neuroendocrine transcription factor ASCL1 (achaete-scute complex homolog-like 1 (ASCL1)) [4]. SCLC is typically highly metastatic in the majority of patients with a large number of circulating tumor cells (CTCs) in the periphery, likely contributing to relapse and poor prognosis [5, 6]. CTCs can have stem cell properties and may help detect early disease, define treatment efficacy or serve as diagnostic markers using liquid biopsy methodology [7–9]. The majority of SCLC patients have a history of smoking and the disease can present itself many years after smoking cessation [10, 11]. SCLC is characterized by rapid and early metastatic growth. SCLC itself is typically not confined to a single tumor mass, instead it spreads rapidly, often as clusters of cancer cells. These clusters or spheroids are commonly observed during metastasis and may contribute to chemoresistance [12]. This suggests that molecules that regulate cell/cell interactions, cell/matrix interactions or in general cytoskeletal functions, are potential therapeutic targets for SCLC.

SCLC is either staged as limited disease (LD), with tumors localized to one hemithorax with potential regional lymph node involvement or ipsilateral pleural effusion, or it is staged as extensive disease (ED) with expanded metastatic states [13]. Most patients are not diagnosed at an early stage, have metastatic disease and face limited treatment options with an overall 5-year survival of less than 7% [14]. It seems apparent that early diagnosis using appropriate biomarkers would improve outcomes. However, to date there are no effective methods for early screening and detection for SCLC. With a 5-year survival rate of less than 20% and more than 30,000 deaths per year, SCLC is defined as a “recalcitrant” cancer in the US. Treatment of SCLC varies depending on the extent of the disease. Standard therapy for LD includes chemotherapy, mostly in combination with radiation. Currently, approximately a quarter of patients with LD can be cured with current standard treatments. Typically, platinum-based chemotherapeutics are combined with etoposide and once or twice daily thoracic radiation therapy before the third cycle of chemotherapy [15–18]. In general, SCLC initially responds well to chemotherapy, yet eventually resistance develops. The median survival for patients diagnosed with LD is 15–20 months and 5.5 months for patients diagnosed with ED. Chemotherapy is also the first-line treatment for patients with ED, but treatment is often palliative [19]. For patients that are not sensitive to etoposide/carboplatin, second-line treatment is mainly limited to the topoisomerase I inhibitor topotecan [20, 21]. Additional therapeutic approaches and targeted therapies have been developed and tested or are under development but have not yet led to a breakthrough in treatment and 5-year overall survival has only marginally improved in the past 40 years [14, 22]. There is thus a clear need to discover new targeted therapies for SCLC and it will be crucial to define biological mechanisms that cause cancer promotion, progression or metastasis and link them to genetic changes. Advances in understanding the biology of SCLC have largely relied on *in vitro* studies using cell line models and may not be suitable for unraveling the sequence of events leading to the aberrations responsible for tumor initiation. Solving these problems remains a challenge, and clinical application of acquired discoveries is often lacking [23, 24].

## SCLC CELLS REPRESENT A DIVERSE POPULATION

Intrinsic and persistent genomic instability in SCLC are a driving force for clonal diversity and disease evolution. With about 175 mutations per tumor, the rate of genomic alterations in SCLC is amongst the highest in solid tumors (5.5 to 7.4 mutations per Mb) [25, 26]. Frequent G-to-T transversions indicate a tobacco carcinogenesis signature and are consistent with a smoking history [25]. Even though SCLC is thought to be of neuroendocrine origin, the cancer stem cell (CSC) population is not well defined and appears diverse on a phenotypical and genomic level, which may be due to high rate of mutations and/or epigenetic regulation. Phenotypically, SCLC CSCs are not well defined and may contain several markers including SOX2, CD44, CD56 (NCAM), CD90, CD105, CD133, Sall4, Oct4, nestin, S100β or vimentin [27, 28]. Interestingly, as the ASCL1 transcription factor regulates neuroendocrine features, it also cooperates with Notch signaling in normal airway stem cell differentiation [29]. In SCLC, this pathway is altered and overexpression of E2F3 as well as loss of RB function drive disease progression, likely supported by loss of function mutations in the tumor suppressor TP53 [30]. Indeed, *TP53* (100%) and *RB1* (93%) are the highest mutated genes in SCLC cases without chromotripsis. [31]. Further, expression of the histone-lysine methyltransferase enhancer of zeste homolog 2 (EZH2) strongly correlated with disruption of E2F transcription factors/RB1 pathway, found in 96% of SCLC [32]. Changes in target gene profiles are also associated with a “stem cell-like” phenotype [32] and in some cases with acquired chemoresistance [33]. *NOTCH* family genes are frequent targets of inactivating mutations in SCLC, with about 25% of tumors being affected and this inactivation is required for optimal tumor growth [31]. In addition, expression of Delta-like protein 3 (DLL3), an inhibitory ligand of NOTCH that is regulated through ASCL1, is expressed in more than 80% of SCLC. Inhibition of DLL3 with rovalpituzumab, a DLL3-targeted antibody-drug conjugate, in recurrent SCLC shows single-agent anti-tumor activity and would be expected to also specifically target CSCs [34]. The overall NOTCH pathway mutational profile in SCLC is consistent with suppression of Notch activity [31]. Additional genes that are mutated at a significant level include the G protein-coupled receptor *FPR1*, the G protein regulatory protein *RGS7* as well as *KIAA1211* and *COL22A1* [31]. There is also amplification of *MYC* family members in about 16% of SCLC [25], which function through transcriptional regulation [35]. There are also alterations in receptor tyrosine kinases, including *FGFR1* and *KIT* or less frequent targets, including *MET, RON and EPHB4* as well as proximal downstream effectors, such as *PIK3CA* or *PTEN* [31, 36–38]. Also, these receptor tyrosine kinases may signal in part through reactive oxygen species (ROS) as a result of metabolic reprogramming that can non-specifically activate signaling cascades and contribute to DNA damage [39]. Metabolic changes in cancer cells are intrinsically associated with transformation and may not only provide energy, but also intermediates for anabolic pathways or metabolites that affect gene expression and ultimately result in changes within the tumor microenvironment, leading to an overall growth advantage. There are additional mechanisms that reflect the genetic heterogeneity within this cancer [25, 31].

## SCLC METASTASIS AND THE TUMOR MICROENVIRONMENT

**Figure 2:**
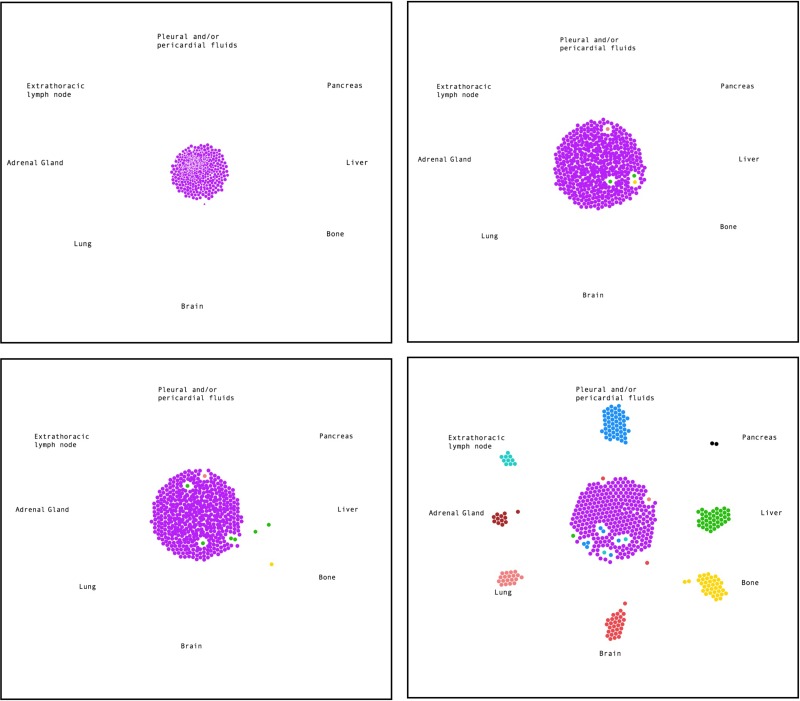
Most common metastasis sites of SCLC Simulation of early time points and the endpoint (from top left to bottom right) of tumor cells (purple) metastasizing to different organs (colored as indicated). See also Supplementary Video 1.

The process of metastasis is divided into several characteristic phases, starting with invasion of tumor cells into surrounding tissues and then eventually the blood stream. As circulating tumor cells (CTCs) they can reach distant sites and grow if they have acquired the capability to survive and interact with the various tissues, such as extravasation through the endothelial lining of blood vessels [40]. The success of this process depends on specific cellular properties that may vary and are not only determined by genomic changes and their cellular consequences but also by the effects that the tumor has on its microenvironment and how it interacts or responds to them. Both extravasion and subsequent intravasion to establish metastatic sites, can be regulated at multiple levels, involving ligands within the extracellular matrix ECM, their receptors, including selectins, integrins, cadherins, CD44 and others, or chemokines and cytokines and their receptors. Additional interaction with immune cells or stromal cells further determine metastatic function [41]. A retrospective study analyzed 251 SCLC patients diagnosed between 1999 and 2000 and found 152 (60.6%) with distant metastases. Target organ involvement included 20.3% liver, 18.3% bone, 15.5% brain, 10.0% lung and 6.0% of adrenal gland [42]. (Figure 2; Supplementary Video 1) The model was generated by javascript bubble chart, where the initial state is fixed with all cancer cells positioned at the primary site, and the final state is quantitatively fixed based on the metastasis sites population reported in Nakazawa *et al*. [42]. Cells with different metastatic phenotypes first appear at the primary site, then cells with similar metastatic phenotypes cluster together and eventually metastasize to the different sites. Metastasis is a multi-step process in the cancer model, we think about mitogenesis, morphogenesis, and motogenesis [43]. The mitogenesis gives the proliferation that also then effects morpho- and moto-genesis. The process of metastasis is dependent on genetic regulation, protein network, and tumor-stroma interaction. As an example, we have shown that PAX5 transcription factor is highly expressed in SCLC [44]. This then regulates chemokine receptor CXCR4 and RTKs such as MET and RON. The receptors themselves cause a plethora of signal transduction events such as activation of the focal adhesion protein FAK and Actin cytoskeleton. Ultimately this leads to increased motility, invasion, and metastasis.

EMT is a crucial process for metastasis formation in cancer progression and development. A few studies have identified small cell lung cancer as an epithelial to mesenchymal transition (EMT)-like cancer [45, 46]. Small cell lung cancer and other carcinomas that undergo EMT have been observed to have an enhanced metastatic capacity, but this is complicated by the presence of epithelial differentiation at the metastatic sites [47]. The forward and backward transitioning between epithelial and mesenchymal phenotypes has become more well understood and it is believed that cancer cells with hybrid phenotypes, in which the EMT network is in a hybrid oscillating state, are associated with more aggressive cancer behaviors [48–50]. In the role of metastasis, it is theorized that the presence of a high number of hybrid E/M cells may form CTC clusters, which form much more metastases than single CTCs [51–53].

The significance of the tumor stromal microenvironment for tumorigenicity and metastasis in SCLC has been well established and some of the mechanisms involved have been already unraveled. In particular, high levels of extracellular matrix (ECM) proteins are found to be specifically associated with SCLC tumors. ECM proteins bind to integrin and other cell surface receptors, effectively regulating cellular transformation processes through ‘outside-in’ signaling. Conversely, ‘inside-out’ signaling regulated by tumor cells defines the interaction of receptors through changes in their functional interaction with ECM proteins [54, 55]. SCLC can actively participate in producing ECM proteins, such as fibronectin and laminin to support anti-apoptotic mechanisms and reduce the efficacy of chemotherapy [56]. Binding of fibronectin to the β1 integrin receptor stimulate PI3K signaling mechanisms that regulate apoptosis, cell motility/morphology and adhesion [56, 57]. Laminin also signals through the PI3K pathway and this activity may be required for suppression of apoptosis, morphological changes and chemoresistance [58]. Invasion and metastasis requires protease activity to penetrate the barrier provided by the ECM. Expression of the ADAM-12 (A disintegrin and metalloprotease-12) protease was found in 73% of tumor specimen and other ADAM family members in 10-40% of specimen. Targeted knockdown of ADAM-12 in the H1688 SCLC cell line reduced cell growth, invasion and metastatic function [59]. Hyaluronic acid, another ECM component, binds mostly to the CD44 receptor and can be cleaved by hyaluronidase. In SCLC, the expression of CD44 and hyaluronidase was found to be low or absent, thus altering potential metastatic function during intravasion and extravasion [60, 61].

The MET receptor, and its ligand, HGF, may also play an important role in metastatic process as well as allowing cancer cells to survive in distant sites [62]. The juxtamembrane R988C and T1010I mutations in MET introduced in a pre-clinical cell line model can lead to growth factor-independent cell growth. Moreover, when overexpressed in the H446 SCLC cell line, both mutations were sufficient to alter cell morphology, adhesion, foci-formation and soft-agar colony formation [38]. MET activation leads in part to phosphorylation and activation of FAK (focal adhesion kinase), AKT, the ERK1/2 pathway and others. Expression of active MET can be targeted by the MET inhibitor, SU11274 or RNA interference, thus regulating MET-dependent invasion [63, 64]. *In vitro* testing of metastatic cells in an orthotropic transplant model showed HGF/MET-dependent motility and invasion of metastatic cells and metastasis itself in bone marrow, kidney and brain could be suppressed by MET inhibition [65]. Ligation of the related receptor tyrosine kinase RON by its ligand macrophage stimulating protein (MSP) suggests a rather specific role in promoting liver metastasis in a SCLC cell line-based *in vivo* mouse model [66]. In patients, there is high expression of the SDF-1/CXCL12 chemokine receptor CXCR4. Normally, SDF-1 is produced in the bone marrow by stromal cells and acts as a homing factor for hematopoietic cells. In SCLC, SDF1 enhances the binding of VCAM-1 (vascular cell adhesion molecule 1 or CD106) to VLA-4 (Very Late Antigen-4 or integrin α4β1). This may explain in part the high propensity of SCLC to metastasize to the bone marrow [67]. This is consistent with an orthotopic xenograph *in vivo* mouse models, wherein tumor growth could be reduced by cisplatin and etoposide, but formation of metastases was not affected. However, the CXCR4 inhibitor AMD3100, was somewhat less effective in reducing tumor growth but significantly reduced suppressed metastasis formation by 43% [68].

The phenotypic plasticity of small cell lung cancer is a dynamic chaotic system where a variety of starting conditions exhibited by molecular interactions and environmental fluctuations evolves into a set of values of the variables, which can be understood as an “attractor” (steady state) where slight perturbations can have robust outcomes [69, 70]. At the core of this system is an endogenous molecular-cellular network that is regulated by gene regulatory networks (GRNs) where fluctuations from normal attractor to cancer attractor are responsible for cancer formation and the eventual cancer progression as cancer attractors transition phenotypes responsible for resistance [70, 71]. A recent study explored the stability of the cancer state that relies on the cancer attractor and the results implied that at a certain state the cancer can no longer be reversed to a non-malignant phenotype [72]. However, existing data shows that it is possible to reverse and maintain aggressive “cancer attractors” by applying a stepwise therapeutic approach by targeting the dynamic system responsible for the primary factors of metastasis and progression [70]. In one example, the transition from normal state occurs through the stepwise process of MDM2 on, CDK2 on, RB off leading to cancer state [72]. However, the reversal processes is similar where RB goes on, CDK2 and CDK4 off, and off of MDM2 leading to a normal state in that sequence [72]. This method of reversing the cancer attractor is vital for SCLC due to the vital role of RB in SCLC oncogenesis, and the restoration of this tumor repressor gene may be a potential therapeutic tactic [72].

As can be appreciated, the genetics/proteomics and growth characteristics for SCLC are unique. In spite of decades of research, we have not made progress in the therapy for SCLC. The role of the immune system in the biology of SCLC is just beginning to emerge and typically depends on the functional interaction of lymphocytes, tumor cells and antigen presenting cells. Currently, several trials determine the efficacy of immune checkpoint inhibitors in SCLC. This treatment is done with a curative intent, since it has the potential to target cancer stem cells, rather than blocking metastatic spread of the tumor [73]. Some of the additional advances for this disease will come from early detection, understanding biology, developing novel therapies, and prevention of recurrence. In order to understand the various genetic and cellular biology of SCLC, we have started to develop mathematical models. In particular, one can evaluate the growth characteristics with first order kinetics such as ordinary differential equations, tumor-stroma interactions with partial differential equations, and geometry/cell movement/metastasis with chaos theory/fractal analysis.

## COMPUTATIONAL MODELING

### Discrete models – cellular automata and agent based models

Single cell events such as mutations have been modeled by discrete methods such as cellular automata [74], agent-based models and hybrid continuum-discrete approaches [75]. These discrete models led to multiscale modeling of cancer where by ordinary differential equations (ODEs) are linked to cellular level parameters [76].

The cellular Potts model [77] is a more generalized cellular automata (CA) that uses lattice dynamics to study interactions among biological cells. The cellular Potts model was used to study the formation of cell clusters as a consequence of assuming configurations of minimal adhesive free energy [78]. The CA model and the cellular Potts model fall under agent based models (ABM). The variables in ABM are individuals. These individuals are considered as agents and a set of prescribed rules govern the behavior of these agents. In the case of CA, the individual lattice cells are the agents.

A discrete agent based spatio-temporal model that incorporates the effects of nutrient supply, mechanical confinement that represents the tissue resistance against tumor cell movement and toxicity of metabolites in the context of brain tumor progression was developed by Mansury *et al*. [79]. They simulated the complex dynamic self-organizing and adaptive processes observed in tumors, namely spatial aggregation of tumor cells as clusters and their migration in search of suitable survival conditions.

Hybrid agent based models combine the unusual effectiveness of continuum deterministic models to capture tumor dynamics at the tissue scale with discrete CA models at cellular and subcellular scales [80]. Tumor invasion of stroma and surrounding tissue are modelled as coupled non-linear partial differential equations (PDEs). The PDEs are discretized to model cell migration and form the basis of the hybrid discrete-continuum model. This model enables specific properties of cells to be described such as proliferation, death, cell-cell adhesion and mutation.

**Figure 3:**
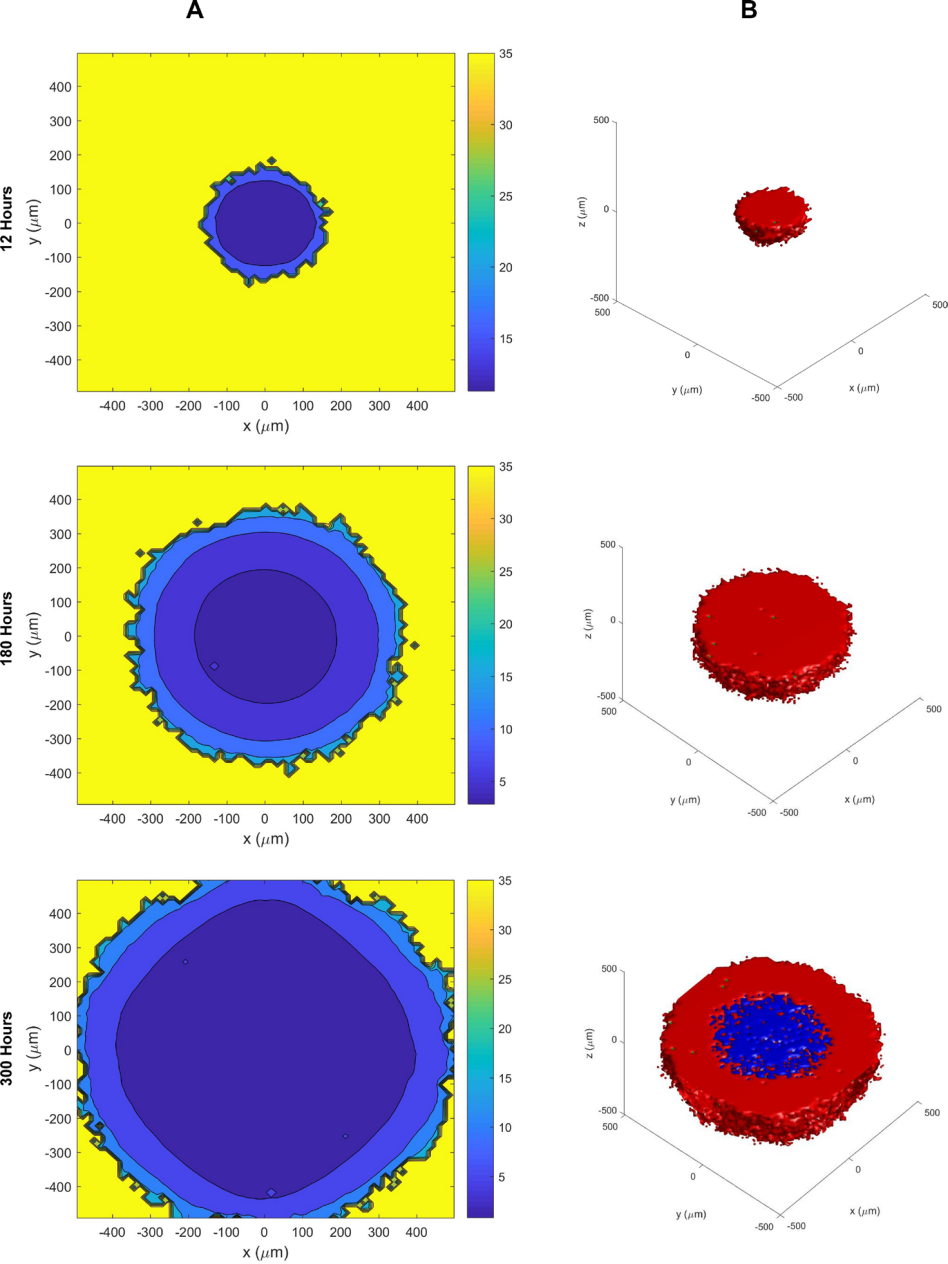
The cellular automaton model of SCLC growth and necrosis (**A**) Growth of the tumor (scale on left side) and oxygen levels within the tumor (color scale on right side) at different time points. See also Supplementary Video 2. (**B**) Growth of the tumor showing the extent of the necrosis at different times (red = live cells, green = apoptotic cells, and blue = necrotic cells). The necrosis of the tumor initiates at > 180 hours. See also Supplementary Video 3.

**Figure 4:**
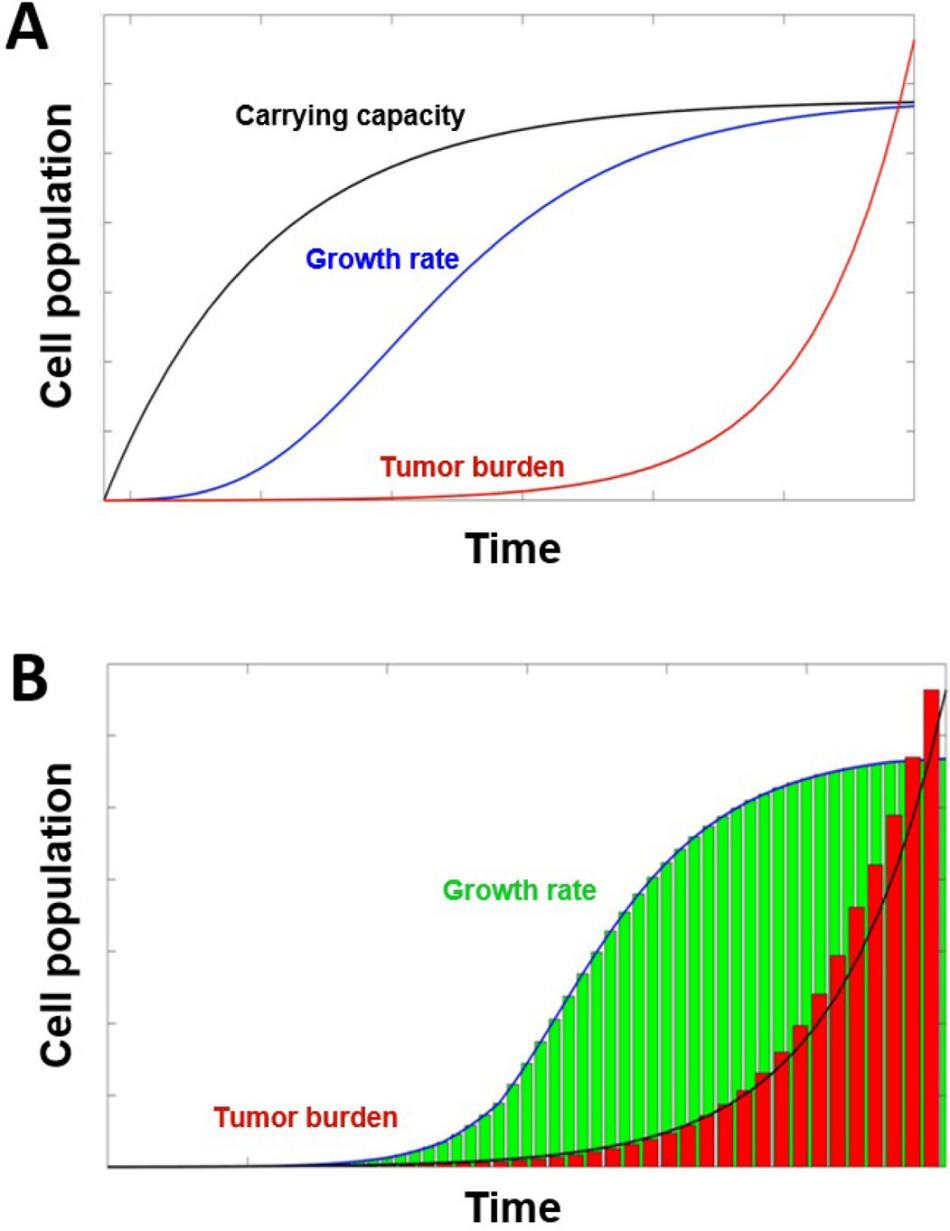
Tumor growth models (**A**) Tumor growth with dynamic carrying capacity and metastatic burden. The growth of the tumor's cell population *c* (blue) and carrying capacity *K* (black) with λ = 0.192, φ = 5.85, and ϕ = 0.00873, parameters used by Enderling and Chaplain [90]. The evolution of metastatic growth rate *d* (red) is presented with the parameters from Benzekry *et al*. [114]. (**B**) Histogram of tumor evolution (green) alongside the histogram (red) of the metastatic growth. As the primary tumor reaches the maximum carrying capacity, the metastatic burden will increase exponentially from the detached primary cells traveling and growing at distant sites (see curve). The metastatic cell population grows beyond the original carrying capacity until it reaches a new carrying capacity due to the model describing the growth of cells in all metastatic sites rather than just the primary site.

The software BioFVM [81] was utilized to simulate the cellular automaton models with some adjustments to values that would favor SCLC (Figure 3, Supplementary Videos 2 and 3). To do this, by increasing the birth rate by .025 and increasing the viable cell Hill coefficient to 4 to simulate our growth rate in Figure 4, modified the viable life span of the tumor cells to increase the probability of apoptosis over time and decrease the time between apoptosis and necrosis due to hypoxia, and introduced a 3% rate of necrosis for perinecrotic tumor cells to fit our carrying capacity obtained in Figure 4. The remaining parameters of the simulation remain similar, where the duration of necrosis and cell death are the same, the system begins with a single cell at time = 0, and the drug parameters are unchanged. The first action performed by the BioFVM program as it creates the simulation is to fill any unoccupied spaces with fluid and considers any dead cells as an empty space [81]. The model then uses the following reaction-diffusion equations for the oxygen and drug, respectively, to apply the uptake rate across each cell:

$$\begin{array}{c} \frac{\partial pO_{2}}{\partial t}=D_{oxy}\nabla^{2}pO_{2}-\lambda_{oxy}pO_{2}-\underset{\text{cells }i}{\sum }U_{i,oxy}pO_{2}\\ \frac{\partial c}{\partial t}=D_{c}\nabla^{2}c-\lambda_{c}c-\underset{\text{cells }i}{\sum }U_{i,c}c \end{array}$$

where the treatment by the drug is set at 5 μM at time *t* = 528 (Day 22). When observing the two-dimensional image of the tumor over time, the cells become more hypoxic the closer they are to the middle. The oxygen concentration in the tumor begins to decrease in steps as the tumor begins to grow, leaving the population of hypoxic cells to grow rapidly.

With the presence of the drug at time *t* = 528, the live tumor cell's exposure to the drug *E* and its response to the drug *R* (having a Hill coefficient h = 1) are given by the equations below:

$$\begin{array}{c} E_{i}\left(t\right)=\overset{t}{\underset{O}{\int }}c\left(s\right)ds\\ R_{i}\left(t\right)=\frac{E_{i}(t)}{\alpha_{i}+E_{i}(t)} \end{array}$$

where α is the exposure for a half-maximum effect [81].

The BioFVM simulations may be captured after the 2-Dimensional tumor profile, cell automata model, basic agent model, and MATLAB model scripts have been edited and saved and the simulations have been run through the command prompt [75]. Once the simulations have finished running, MATLAB is automatically prompted and a series of images, or visual interpretations of the system at each recorded time interval, are then opened. These images may then be saved as image files. This spatio-temporal model could be utilized to predict and personalize patient response to drug therapy using organoids, spheroids, mouse models, and zebrafish models.

### Discrete models – cellular automata, tumor microenvironment, and cell viability

Cancer models based on differential equations address a continuum of cells at the tissue scale where the effect of individual cells is averaged. On the other hand, discrete models of tumor growth based on CA capture the response of individual cells as they interact with one another as well as with its microenvironment. CA is a collection of cells arranged on a lattice that evolve with time according to a defined set of rules that includes the values of neighboring cells. The first practical application of CA was shown by John Conway in “Game of Life” [82]. CA models have been used to study tumor growth at the cellular scale [83–87] and subcellular scale [88, 89]. For example, CA was used to study the invasion of cancerous cells in a population of normal cells by Qi *et al*. [86]. In this context, a lattice cell represented a single biological cell. The state of each of the cells of the CA was assumed to be normal, cancerous, complex (cancerous and bound by white blood cell) or dead cancerous. Probabilistic rules were then applied to study the dynamics of the cellular states. However, this model did not explicitly consider growth promoting factors (such as presence of blood vessels, nutrient supply and oxygen) and growth inhibiting factors (such as toxic metabolites) for tumors that ‘motivate’ them to move far away from primary sites.

The microenvironment-dependent birth rate *b_i_* and death rate d_*i*_ are then modeled how Macklin *et al.* described as shown below:

$$\begin{array}{c} b_{i}\left(t\right)=\;\{\begin{array}{cc} b_{i,P}\left(1-\eta_{i}R_{i}\left(t\right)\right) & \text{if}\;pO_{2,P}<pO_{2}\\ b_{i,P}\left(\frac{pO_{2}-pO_{2,N}}{pO_{2,P}-pO_{2,N}}\right)\left(1-\eta_{i}R_{i}\left(t\right)\right) & \text{if }pO_{2,N}<pO_{2}\leq pO_{2,P}\\ 0 & \text{if }pO_{2}\leq pO_{2,N} \end{array}\\ d_{i}\left(t\right)=\;\{\begin{array}{cc} 0 & \text{if }pO_{2,N}<pO_{2}\\ d_{i,N}^{*} & \text{if }pO_{2}\leq pO_{2,N} \end{array} \end{array}$$

where d^*^_i,N_ is a constant [81]. The microenvironment apoptosis rate *d_i,A_* is modeled as:

$$d_{i,A}\left(t\right)=d_{i,A}^{*}+(d_{i,A}^{max}-d_{i,A}^{*})R_{i}(t)$$

where *d^max^* is the maximum rate of apoptosis [81].

For the time interval [t, t+Δt], each viable tumor cell has the chance to divide, undergo apoptotic death, or reach necrotic death. The probability for a live cell to perform one of the three actions in each time step until death is described by the equations below [81]:

$$\begin{array}{c} P\left(\text{cell division}\right)=1-\exp(d_{i,A}\left(t\right)\mathrm{\Delta t})\\ P\left(\text{apoptotic death}\right)=1-\exp(b_{i}\left(t\right)\mathrm{\Delta t})\\ P\left(\text{necrotic death}\right)=1-\exp(-d_{i,N}\left(t\right)\mathrm{\Delta t}) \end{array}$$

Each dead cell then has the probability that it will become lytic and rupture, leaving an empty space in the automaton model, based on the average duration the cell death. The probability of the dead cell to reach lysis is expressed in the equation below where T_D_ is the duration of cell death [81]:

$$P\left(\text{cell lysis}\right)=1-\exp(-\frac{1}{T_{i,D}}\Delta t)$$

### Continuous models − ODEs and PDEs

**Figure 5:**
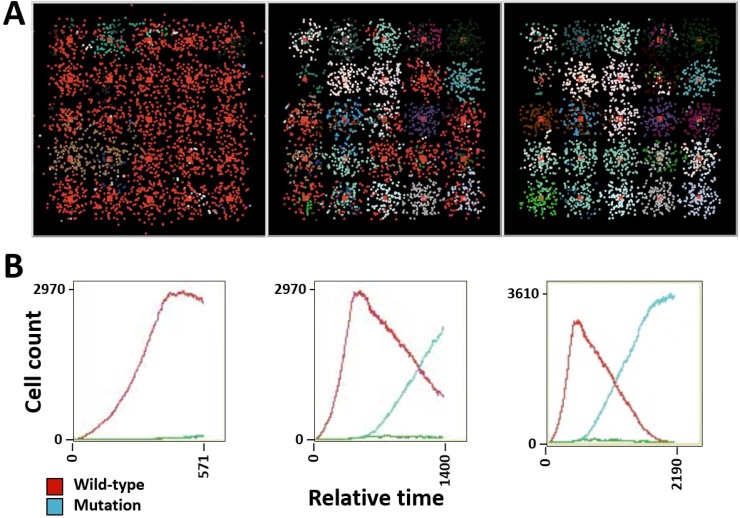
Simulation of tumor heterogeneity and tumor burden in SCLC (**A**) Tumor heterogeneity and cellular automata at different time points. (**B**) Tumor burden at corresponding time points, indicating the growth of wild-type cells (red) and cells containing mutations (blue). See also Supplementary Video 4.

For a given set of initial conditions, models that produce the same results each time they are solved are known as deterministic models. These differ from stochastic or probabilistic models in that the model results change each time they are solved even though the initial conditions don't. Deterministic models with one independent variable (“time”) and one or more dependent variables (such as “substrates” or “metabolites”) and represented by ODEs are ideal to capture dynamical processes. For example, Enderling and Chaplain [90] studied the rate of tumor growth cells. Utilizing parameters α (fraction of dividing tumor cells) and β (fraction of tumor dying cells), they showed that tumor cells could either be in 1) quiescence (α – β = 0), 2) proliferative (α > β) or 3) depleting (α < β). Since tumors do not grow indefinitely in size, a more realistic representation of rate of tumor growth should take into account the carrying capacity constraint, K, representing the maximum population of cells also known as carrying capacity of the host cell. Hahnefeldt and colleagues [91] modelled K as a function of time and tumor size as follows:

$$\frac{\text{dK}}{\text{dt}}=\phi C-\;\varphi C^{\frac{2}{3}}$$

where ϕ and φ represent constant positive rates of angiogenesis stimulation and inhibition, respectively (Figures 4 and 5, Supplementary Video 4).

Mathematical models solely based on ODEs describe the total number of tumor cells over time but do not consider any spatial variables. It is essential to model the spatial variables along with time since processes such as cancer invasion and metastases are more potent killers than local tumor growth and are inherently spatial in nature. Models based on PDEs such as reaction diffusion are apt for quantitative substances of interest in cancer modeling (such as nutrients or oxygen) at a specific position (space) and time (t). PDE based models are also referred to as continuum models since they are solved for continuously in space and time variables.

For example, Gatenby and Gawlinski [92] were one of the earliest to model cancer invasion as a spatio-temporal evolution of tumor cells (C), enzymes with H+ ions (m) and extracellular matrix (υ) as follows:

$$\begin{array}{c} \frac{\partial C}{\partial t}=\nabla .\left({\text{D}}_{C}\;\left(1-\upsilon \right)\nabla C\right)+\rho C\left(1-C\right)\\ \frac{\partial \text{m}}{\partial t}=\nabla^{2}\text{m}+\delta \left(C-\text{m}\right)\\ \frac{\partial \upsilon }{\partial t}=\upsilon \left(1-\upsilon \right)-\gamma \text{m}\upsilon \end{array}$$

where D_C_ is the diffusion coefficient constant, ρ is the tumor cell proliferation rate constant, δ is the H+ ions production and decay rate constant and γ is the extracellular matrix degradation rate. Appropriate initial conditions and spatial region need to be specified to solve $\frac{\partial \text{m}}{\partial \text{t}}=\nabla^{2}\text{m}+\delta \left(\text{C}-\text{m}\right)$ where tumor cells are assumed to proliferate and undergo nonlinear diffusion and secrete H+ ions which diffuse and degrade the normal tissue. The H+ ions are assumed to undergo linear decay with logistic growth for normal tissue in the absence of any cancer cells. Cancer cell migration processes such as haptotaxis (i.e. directional cell migration in response to gradients of cellular adhesion molecules in the extracellular matrix or gradients of the extracellular matrix density) were modeled using a modified version of $\frac{\partial \text{m}}{\partial \text{t}}=\nabla^{2}\text{m}+\delta \left(\text{C}-\text{m}\right)$ by Anderson *et al*. [93].

The PDE based models can be discretized using finite-difference approximations. To study individual cell movement, Anderson *et al*. investigated the discrete form of the continuous version built to study haptotaxis [94]. Spatial variables were discretized retaining time, t, to be continuous. Stochastic movement rules were incorporated to derive a biased random walk governing the motion of a single tumor cell. Dynamical models of cancer growth leading to chaotic behavior have also been reported [95]. Itik and Banks were able to explicitly show the existence of deterministic chaotic dynamics by modeling the interactions and competitions between tumor cells and other cells of the body such as healthy host cells and activated immune system cells. Based on ideas from Lie algebra [96], the control of chaotic dynamics of cancer growth has been recently formulated [97] in a three dimension cancer model (TDCM) for tumor growth. This spatio-temporal heterogeneity model could be utilized to understand the tumor evolution over time as well as attempt to predict the genetic phenotype that may correlate with metastasis and cancer progression. As we go forward from here, we have to be able to incorporate mathematical modeling in SCLC. We can envision the utilization of the various models in the behavior of cell lines/three-dimensional models, organoids/spheroids along with PDX/CDX models, as well as tumor behavior in natural progression and/or therapeutic response. In particular, we should be able to study the potential for mechanisms of resistance.

### MultiFractals – applications to image data analysis

Most naturally occurring phenomena exhibit complexity and irregularity in structure. Classical techniques in mathematics are not adept at capturing the underlying geometry. Irregular objects for which the “Hausdorff dimension” is strictly greater than the “Topological dimension” were defined as fractals by Benoit B. Mandelbrot [98]. Even though fractal objects seem to have complex shapes and exhibit dynamic behavior, they always exhibit a nesting of statistically self-similar features at different scales [99]. The branch of a tree has similar structure to the tree but at a smaller scale. A high degree of self-similarity has also been documented in human tissue [100]. Fractals are compared by their fractal dimension, a real number, which is a measure of the size of the underlying fractal sets. There are multiple methods to compute the fractal dimension. The similarity dimension [101], D_S_ is ideal for computing the fractal dimension of artificially generated fractals (following precise creation rules and having regular structure). It is defined as follows:

$$D_{S}=-\frac{\ln N}{\ln r}$$

where N represents the number of non-overlapping copies of the object and r (< 1) represents the scaling ratio. The box counting dimension [102] that is most widely used in practice to compute the fractal dimension takes into account the relationship between non-empty boxes and box-size necessary to cover the fractal object. The fractal dimensions of most objects found in nature are best computed using the box counting method. The fractal dimension has been applied as one of the features for classifying pathological tissue in mammograms and tumor blood vessels [103, 104].

The box counting approach to computing the fractal dimension assumes that the fractal object can completely be described by two states (presence inside or outside the box). However, most natural phenomena such as greyscale image of tissue or cells require the intensity inside a box to be considered as well. Since the intensity levels of such an image can be non-uniform resulting in low to high densities, their structure characterized by their fractal dimension would vary with the observed scale.

Unlike synthetically generated fractals, the structure of naturally occurring fractal objects at different scales will be similar but not exactly same as the whole. This results in the co-existence of multiple fractal subsets with different scaling behavior. Therefore, instead of one set of fractals we are typically faced with multifractals.

Multifractal analysis requires the description of a local and global measure over a region. For each point of the region, the local singularity coefficient (i.e. the local dimension) is known as the “Holder exponent” or α values [101, 102]. In image data analysis, α values reflect the local behavior of the intensity measure that are of 4 different types [105, 106]. The corresponding image is often referred to as the α –image. A region would contain a range of positive, finite α values, with a minimum value, α_min_ and a maximum value, α_max_. The fractal dimension can be computed using the box counting method [107] over a set of points (isolated or otherwise) having the same α value. A plot of fractal dimension against α values results in a multifractal spectrum, f(α) (i.e. the global measure) after adjusting for noise in digital images.

Multifractal theory has been applied in the context of medical image data analysis [103] and object classification [101]. Using multifractal analysis of breast cancer tissue prior to chemotherapy Vasiljevic *et al*. [107] showed that the tissue can be differentiated based on their sensitivity to chemotherapy. Small cell lung cancer embodies the essence of the aberrant tumor cell which despite its diverse genotype has a distinctive, discrete, and easily recognizable tumor tissue [69]. These unique spatial characteristics could be characterized utilizing the multifractal model at different scaling magnifications such as DNA random walks, tumor tissue, and radiological scans.

## CONCLUSIONS

We have summarized and applied several mathematical models to SCLC that enable us to replicate some of its unique fractal characteristics. Features of SCLC tumors are a reflection of alterations in genes associated with the disease including overexpression, somatic mutation and amplification, resulting in a plethora of targeted therapies existing or in development including FAK inhibitors, RTK inhibitors targeting KIT, IGF-1R, EPHB4, RON and MET as well as their downstream pathways including the PI3K/AKT/mTOR and MYC [108]. Further areas for drug therapies in SCLC include the apoptotic pathway, the hedgehog and DNA repair pathways, heat shock proteins and HDAC as well as angiogenesis pathways and others [109]. Additional novel therapies in development include targeting of cancer stem cells and immunotherapy [110]. Despite these targeted therapies, the prognosis for SCLC remains grim [111, 112]. Most recently, there have been immunotherapies that have come to fruition. Checkpoint inhibition appears to work with pembrolizumab or nivolumab/ipilimumab. There are specific other programs, such as bispecific antibodies and CAR-T cells that are just beginning to be employed. The effective utilization of mathematical models to these therapies is hampered in part by the availability of quantitative *in vitro* experimental data and clinical results. Whereas the goal remains to link outcomes to the fractalness of SCLC, these techniques may be particularly useful in the context of drug development research, particularly in combination with existing research platforms, such as various omics approaches that provide large amounts of data. As clinical results can vary widely, dependent on the patient's specific tumor alterations, it will be important to link them to these genetic changes. Even though the models presented here are not perfect, they provide us with the tools to allow us to breakdown the mechanisms of growth and metastasis and eventually add additional parameters. We can really state that models depend on the circumstance. ODE can be utilized to study tumor kinetics, PDE on tumor-stroma interactions, and chaos theory on geometry and symmetry breaking [113] in metastasis. The complexity of any physical or biological system can be quantified in terms of combinatorial, geometric and functional components. Each of these components is best characterized by their symmetries. Symmetric breaking occurs when the natural symmetry is broken either explicitly or spontaneously. Understanding cancer processes like metastasis as a sequence of symmetry breaking events will be required. The fractal analysis gives us a unique insight on geometry and ultimately could serve as a biomarker. An important goal is to link the existing comprehensive set of analytic tools back to the fractalness of cancer and provide a platform for accurate biomarker development. The identification of appropriate biomarkers in combination with mathematical modeling has the potential to provide breakthroughs in the development of therapeutics. Since SCLC is a rapidly growing disease, it will be important to utilize these powerful mathematical tools to enhance our understanding of the biology of this devastating cancer and ultimately accelerating the development of novel therapeutics.

## SUPPLEMENTARY MATERIALS VIDEOS











## Abbreviations

SCLC: small cell lung cancer
ASCL1: achaete-scute complex homolog-like 1
LD: limited disease
ED: extensive disease
Mb: Megabase
CSC: cancer stem cell
SOX2: Sex Determining Region Y-Box 2
Sall4: Spalt like transcription factor 4
Oct4: Octamer-binding transcription factor 4
E2F3: E2F transcription factor 3
TP53: Tumor protein p53
RB1: RB transcriptional compressor 1
NCAM: Neural cell adhesion molecule or CD56
EZH2: enhancer of zeste homolog 2
DLL3: Delta-like protein 3
FPR1: formyl peptide receptor 1
RGS7: regulator of G protein signaling 7
COL22A1: Collagen Type XXII Alpha I Chain
MYC: V-Myc Avian Myelocytomatosis Viral Oncogene Homolog
MET: hepatocyte growth factor receptor
FGFR1: Fibroblast growth factor receptor 1
KIT: V-Kit Hardy-Zuckerman 4 feline sarcoma viral oncogene
EPHB4: EPH receptor B4
PIK3CA: Phosphatidylinositol-4, 5-Bisphosphate 3-Kinase Catalytic Subunit Alpha
PTEN: Phosphatase and Tensin homolog
RON: Recepteur d'Origine Nantais
MST1R: Macrophage stimulating 1 receptor
ROS: reactive oxygen species
CTCs: circulating tumor cells
ECM: extracellular matrix
PI3K: phosphoinositide 3-kinase
ADAM-12: A disintegrin and metalloprotease-12
FAK: focal adhesion kinase
AKT: Protein kinase B
ERK: extracellular signal-regulated kinase
MSP: macrophage stimulating protein
MSP: macrophage stimulating protein
VCAM-1: vascular cell adhesion molecule 1 or CD106
VLA-4: Very Late Antigen 4 or integrin α4β1
CXCR4: C-X-C Motif Chemokine Receptor 4
CA: cellular automata
ODE: ordinary differential equations
PDE: partial differential equations
ABM: agent based models
BioFVM: Finite Volume Method for biological problems
RTK: receptor tyrosine kinase
HDAC: histone deacetylase
CAR-T: chimeric antigen receptor T

## References

[1] Park KS, Liang MC, Raiser DM, Zamponi R, Roach RR, Curtis SJ, Walton Z, Schaffer BE, Roake CM, Zmoos AF, Kriegel C, Wong KK, Sage J (2011). Characterization of the cell of origin for small cell lung cancer. Cell Cycle.

[2] Lantuejoul S, Moro D, Michalides RJ, Brambilla C, Brambilla E (1998). Neural cell adhesion molecules (NCAM) and NCAM-PSA expression in neuroendocrine lung tumors. Am J Surg Pathol.

[3] Patel K, Moore SE, Dickson G, Rossell RJ, Beverley PC, Kemshead JT, Walsh FS (1989). Neural cell adhesion molecule (NCAM) is the antigen recognized by monoclonal antibodies of similar specificity in small-cell lung carcinoma and neuroblastoma. Int J Cancer.

[4] Borges M, Linnoila RI, van de Velde HJ, Chen H, Nelkin BD, Mabry M, Baylin SB, Ball DW (1997). An achaete-scute homologue essential for neuroendocrine differentiation in the lung. Nature.

[5] Hodgkinson CL, Morrow CJ, Li Y, Metcalf RL, Rothwell DG, Trapani F, Polanski R, Burt DJ, Simpson KL, Morris K, Pepper SD, Nonaka D, Greystoke A (2014). Tumorigenicity and genetic profiling of circulating tumor cells in small-cell lung cancer. Nat Med.

[6] Yu N, Zhou J, Cui F, Tang X (2015). Circulating tumor cells in lung cancer: detection methods and clinical applications. Lung.

[7] Carter L, Rothwell DG, Mesquita B, Smowton C, Leong HS, Fernandez-Gutierrez F, Li Y, Burt DJ, Antonello J, Morrow CJ, Hodgkinson CL, Morris K, Priest L (2017). Molecular analysis of circulating tumor cells identifies distinct copy-number profiles in patients with chemosensitive and chemorefractory small-cell lung cancer. Nat Med.

[8] Hou JM, Krebs MG, Lancashire L, Sloane R, Backen A, Swain RK, Priest LJ, Greystoke A, Zhou C, Morris K, Ward T, Blackhall FH, Dive C (2012). Clinical significance and molecular characteristics of circulating tumor cells and circulating tumor microemboli in patients with small-cell lung cancer. J Clin Oncol.

[9] Tanaka F, Yoneda K, Kondo N, Hashimoto M, Takuwa T, Matsumoto S, Okumura Y, Rahman S, Tsubota N, Tsujimura T, Kuribayashi K, Fukuoka K, Nakano T (2009). Circulating tumor cell as a diagnostic marker in primary lung cancer. Clin Cancer Res.

[10] Ou SH, Ziogas A, Zell JA (2009). Prognostic factors for survival in extensive stage small cell lung cancer (ED-SCLC): the importance of smoking history, socioeconomic and marital statuses, and ethnicity. J Thorac Oncol.

[11] Varghese AM, Zakowski MF, Yu HA, Won HH, Riely GJ, Krug LM, Kris MG, Rekhtman N, Ladanyi M, Wang L, Berger MF, Pietanza MC (2014). Small-cell lung cancers in patients who never smoked cigarettes. J Thorac Oncol.

[12] Klameth L, Rath B, Hochmaier M, Moser D, Redl M, Mungenast F, Gelles K, Ulsperger E, Zeillinger R, Hamilton G (2017). Small cell lung cancer: model of circulating tumor cell tumorospheres in chemoresistance. Sci Rep.

[13] Shepherd FA, Crowley J, Van Houtte P, Postmus PE, Carney D, Chansky K, Shaikh Z, Goldstraw P, International Association for the Study of Lung Cancer International Staging Committee and Participating Institutions (2007). The International Association for the Study of Lung Cancer lung cancer staging project: proposals regarding the clinical staging of small cell lung cancer in the forthcoming (seventh) edition of the tumor, node, metastasis classification for lung cancer. J Thorac Oncol.

[14] Wang S, Tang J, Sun T, Zheng X, Li J, Sun H, Zhou X, Zhou C, Zhang H, Cheng Z, Ma H, Sun H (2017). Survival changes in patients with small cell lung cancer and disparities between different sexes, socioeconomic statuses and ages. Sci Rep.

[15] Gregor A, Drings P, Burghouts J, Postmus PE, Morgan D, Sahmoud T, Kirkpatrick A, Dalesio O, Giaccone G (1997). Randomized trial of alternating versus sequential radiotherapy/chemotherapy in limited-disease patients with small-cell lung cancer: a European Organization for Research and Treatment of Cancer Lung Cancer Cooperative Group Study. J Clin Oncol.

[16] Jeremic B, Shibamoto Y, Acimovic L, Milisavljevic S (1997). Initial versus delayed accelerated hyperfractionated radiation therapy and concurrent chemotherapy in limited small-cell lung cancer: a randomized study. J Clin Oncol.

[17] Murray N, Coy P, Pater JL, Hodson I, Arnold A, Zee BC, Payne D, Kostashuk EC, Evans WK, Dixon P, Sadura A, Feld R, Levitt M (1993). Importance of timing for thoracic irradiation in the combined modality treatment of limited-stage small-cell lung cancer. The National Cancer Institute of Canada Clinical Trials Group. J Clin Oncol.

[18] Takada M, Fukuoka M, Kawahara M, Sugiura T, Yokoyama A, Yokota S, Nishiwaki Y, Watanabe K, Noda K, Tamura T, Fukuda H, Saijo N (2002). Phase III study of concurrent versus sequential thoracic radiotherapy in combination with cisplatin and etoposide for limited-stage small-cell lung cancer: results of the Japan Clinical Oncology Group Study 9104. J Clin Oncol.

[19] Alvarado-Luna G, Morales-Espinosa D (2016). Treatment for small cell lung cancer, where are we now?-a review. Transl Lung Cancer Res.

[20] Eckardt JR, von Pawel J, Pujol JL, Papai Z, Quoix E, Ardizzoni A, Poulin R, Preston AJ, Dane G, Ross G (2007). Phase III study of oral compared with intravenous topotecan as second-line therapy in small-cell lung cancer. J Clin Oncol.

[21] O'Brien ME, Ciuleanu TE, Tsekov H, Shparyk Y, Cucevia B, Juhasz G, Thatcher N, Ross GA, Dane GC, Crofts T (2006). Phase III trial comparing supportive care alone with supportive care with oral topotecan in patients with relapsed small-cell lung cancer. J Clin Oncol.

[22] Ma PC, Salgia R (2004). Novel targets for therapeutic agents in small cell lung cancer. J Natl Compr Canc Netw.

[23] Bharti A, Ma PC, Maulik G, Singh R, Khan E, Skarin AT, Salgia R (2004). Haptoglobin alpha-subunit and hepatocyte growth factor can potentially serve as serum tumor biomarkers in small cell lung cancer. Anticancer Res.

[24] Poroyko V, Mirzapoiazova T, Nam A, Mambetsariev I, Mambetsariev B, Wu X, Husain A, Vokes EE, Wheeler DL, Salgia R (2018). Exosomal miRNAs species in the blood of small cell and non-small cell lung cancer patients. Oncotarget.

[25] Peifer M, Fernandez-Cuesta L, Sos ML, George J, Seidel D, Kasper LH, Plenker D, Leenders F, Sun R, Zander T, Menon R, Koker M, Dahmen I (2012). Integrative genome analyses identify key somatic driver mutations of small-cell lung cancer. Nat Genet.

[26] Rudin CM, Durinck S, Stawiski EW, Poirier JT, Modrusan Z, Shames DS, Bergbower EA, Guan Y, Shin J, Guillory J, Rivers CS, Foo CK, Bhatt D (2012). Comprehensive genomic analysis identifies SOX2 as a frequently amplified gene in small-cell lung cancer. Nat Genet.

[27] Codony-Servat J, Verlicchi A, Rosell R (2016). Cancer stem cells in small cell lung cancer. Transl Lung Cancer Res.

[28] Zhang Z, Zhou Y, Qian H, Shao G, Lu X, Chen Q, Sun X, Chen D, Yin R, Zhu H, Shao Q, Xu W (2013). Stemness and inducing differentiation of small cell lung cancer NCI-H446 cells. Cell Death Dis.

[29] Ball DW (2004). Achaete-scute homolog-1 and Notch in lung neuroendocrine development and cancer. Cancer Lett.

[30] Kitamura H, Yazawa T, Sato H, Okudela K, Shimoyamada H (2009). Small cell lung cancer: significance of RB alterations and TTF-1 expression in its carcinogenesis, phenotype, and biology. Endocr Pathol.

[31] George J, Lim JS, Jang SJ, Cun Y, Ozretic L, Kong G, Leenders F, Lu X, Fernandez-Cuesta L, Bosco G, Muller C, Dahmen I, Jahchan NS (2015). Comprehensive genomic profiles of small cell lung cancer. Nature.

[32] Coe BP, Thu KL, Aviel-Ronen S, Vucic EA, Gazdar AF, Lam S, Tsao MS, Lam WL (2013). Genomic deregulation of the E2F/Rb pathway leads to activation of the oncogene EZH2 in small cell lung cancer. PLoS One.

[33] Gardner EE, Lok BH, Schneeberger VE, Desmeules P, Miles LA, Arnold PK, Ni A, Khodos I, de Stanchina E, Nguyen T, Sage J, Campbell JE, Ribich S (2017). Chemosensitive Relapse in Small Cell Lung Cancer Proceeds through an EZH2-SLFN11 Axis. Cancer Cell.

[34] Rudin CM, Pietanza MC, Bauer TM, Ready N, Morgensztern D, Glisson BS, Byers LA, Johnson ML, Burris HA, Robert F, Han TH, Bheddah S, Theiss N (2017). Rovalpituzumab tesirine, a DLL3-targeted antibody-drug conjugate, in recurrent small-cell lung cancer: a first-in-human, first-in-class, open-label, phase 1 study. Lancet Oncol.

[35] Conacci-Sorrell M, McFerrin L, Eisenman RN (2014). An overview of MYC and its interactome. Cold Spring Harb Perspect Med.

[36] Ferguson BD, Tan YH, Kanteti RS, Liu R, Gayed MJ, Vokes EE, Ferguson MK, Iafrate AJ, Gill PS, Salgia R (2015). Novel EPHB4 Receptor Tyrosine Kinase Mutations and Kinomic Pathway Analysis in Lung Cancer. Sci Rep.

[37] Kanteti R, Krishnaswamy S, Catenacci D, Tan YH, EL-Hashani E, Cervantes G, Husain AN, Tretiakova M, Vokes EE, Huet H, Salgia R (2012). Differential expression of RON in small and non-small cell lung cancers. Genes Chromosomes Cancer.

[38] Ma PC, Kijima T, Maulik G, Fox EA, Sattler M, Griffin JD, Johnson BE, Salgia R (2003). c-MET mutational analysis in small cell lung cancer: novel juxtamembrane domain mutations regulating cytoskeletal functions. Cancer Res.

[39] Jagadeeswaran R, Jagadeeswaran S, Bindokas VP, Salgia R (2007). Activation of HGF/c-Met pathway contributes to the reactive oxygen species generation and motility of small cell lung cancer cells. Am J Physiol Lung Cell Mol Physiol.

[40] Micalizzi DS, Maheswaran S, Haber DA (2017). A conduit to metastasis: circulating tumor cell biology. Genes Dev.

[41] Reymond N, d'Agua BB, Ridley AJ (2013). Crossing the endothelial barrier during metastasis. Nat Rev Cancer.

[42] Nakazawa K, Kurishima K, Tamura T, Kagohashi K, Ishikawa H, Satoh H, Hizawa N (2012). Specific organ metastases and survival in small cell lung cancer. Oncol Lett.

[43] Lukas RV, Gondi V, Kamson DO, Kumthekar P, Salgia R (2017). State-of-the-art considerations in small cell lung cancer brain metastases. Oncotarget.

[44] Kanteti R, Nallasura V, Loganathan S, Tretiakova M, Kroll T, Krishnaswamy S, Faoro L, Cagle P, Husain AN, Vokes EE, Lang D, Salgia R (2009). PAX5 is expressed in small-cell lung cancer and positively regulates c-Met transcription. Lab Invest.

[45] Krohn A, Ahrens T, Yalcin A, Plones T, Wehrle J, Taromi S, Wollner S, Follo M, Brabletz T, Mani SA, Claus R, Hackanson B, Burger M (2014). Tumor cell heterogeneity in Small Cell Lung Cancer (SCLC): phenotypical and functional differences associated with Epithelial-Mesenchymal Transition (EMT) and DNA methylation changes. PLoS One.

[46] Ito T, Kudoh S, Ichimura T, Fujino K, Hassan WA, Udaka N (2017). Small cell lung cancer, an epithelial to mesenchymal transition (EMT)-like cancer: significance of inactive Notch signaling and expression of achaete-scute complex homologue 1. Hum Cell.

[47] Calbo J, van Montfort E, Proost N, van Drunen E, Beverloo HB, Meuwissen R, Berns A (2011). A functional role for tumor cell heterogeneity in a mouse model of small cell lung cancer. Cancer Cell.

[48] Lu M, Jolly MK, Gomoto R, Huang B, Onuchic J, Ben-Jacob E (2013). Tristability in cancer-associated microRNA-TF chimera toggle switch. J Phys Chem B.

[49] Lu M, Jolly MK, Levine H, Onuchic JN, Ben-Jacob E (2013). MicroRNA-based regulation of epithelial-hybrid-mesenchymal fate determination. Proc Natl Acad Sci U S A.

[50] George JT, Jolly MK, Xu S, Somarelli JA, Levine H (2017). Survival Outcomes in Cancer Patients Predicted by a Partial EMT Gene Expression Scoring Metric. Cancer Res.

[51] Aceto N, Bardia A, Miyamoto DT, Donaldson MC, Wittner BS, Spencer JA, Yu M, Pely A, Engstrom A, Zhu H, Brannigan BW, Kapur R, Stott SL (2014). Circulating tumor cell clusters are oligoclonal precursors of breast cancer metastasis. Cell.

[52] Bottos A, Hynes NE (2014). Cancer: Staying together on the road to metastasis. Nature.

[53] Cheung KJ, Padmanaban V, Silvestri V, Schipper K, Cohen JD, Fairchild AN, Gorin MA, Verdone JE, Pienta KJ, Bader JS, Ewald AJ (2016). Polyclonal breast cancer metastases arise from collective dissemination of keratin 14-expressing tumor cell clusters. Proc Natl Acad Sci U S A.

[54] Buttery RC, Rintoul RC, Sethi T (2004). Small cell lung cancer: the importance of the extracellular matrix. Int J Biochem Cell Biol.

[55] Rintoul RC, Sethi T (2001). The role of extracellular matrix in small-cell lung cancer. Lancet Oncol.

[56] Sethi T, Rintoul RC, Moore SM, MacKinnon AC, Salter D, Choo C, Chilvers ER, Dransfield I, Donnelly SC, Strieter R, Haslett C (1999). Extracellular matrix proteins protect small cell lung cancer cells against apoptosis: a mechanism for small cell lung cancer growth and drug resistance in vivo. Nat Med.

[57] Kijima T, Maulik G, Ma PC, Madhiwala P, Schaefer E, Salgia R (2003). Fibronectin enhances viability and alters cytoskeletal functions (with effects on the phosphatidylinositol 3-kinase pathway) in small cell lung cancer. J Cell Mol Med.

[58] Tsurutani J, West KA, Sayyah J, Gills JJ, Dennis PA (2005). Inhibition of the phosphatidylinositol 3-kinase/Akt/mammalian target of rapamycin pathway but not the MEK/ERK pathway attenuates laminin-mediated small cell lung cancer cellular survival and resistance to imatinib mesylate or chemotherapy. Cancer Res.

[59] Shao S, Li Z, Gao W, Yu G, Liu D, Pan F (2014). ADAM-12 as a diagnostic marker for the proliferation, migration and invasion in patients with small cell lung cancer. PLoS One.

[60] Junker N, Latini S, Petersen LN, Kristjansen PE (2003). Expression and regulation patterns of hyaluronidases in small cell lung cancer and glioma lines. Oncol Rep.

[61] Richter U, Wicklein D, Geleff S, Schumacher U (2012). The interaction between CD44 on tumour cells and hyaluronan under physiologic flow conditions: implications for metastasis formation. Histochem Cell Biol.

[62] Lawrence RE, Salgia R (2010). MET molecular mechanisms and therapies in lung cancer. Cell Adh Migr.

[63] Ma PC, Tretiakova MS, Nallasura V, Jagadeeswaran R, Husain AN, Salgia R (2007). Downstream signalling and specific inhibition of c-MET/HGF pathway in small cell lung cancer: implications for tumour invasion. Br J Cancer.

[64] Wang ZX, Lu BB, Yang JS, Wang KM, De W (2011). Adenovirus-mediated siRNA targeting c-Met inhibits proliferation and invasion of small-cell lung cancer (SCLC) cells. J Surg Res.

[65] Sakamoto S, Inoue H, Ohba S, Kohda Y, Usami I, Masuda T, Kawada M, Nomoto A (2015). New metastatic model of human small-cell lung cancer by orthotopic transplantation in mice. Cancer Sci.

[66] Sato S, Hanibuchi M, Kuramoto T, Yamamori N, Goto H, Ogawa H, Mitsuhashi A, Van TT, Kakiuchi S, Akiyama S, Nishioka Y, Sone S (2013). Macrophage stimulating protein promotes liver metastases of small cell lung cancer cells by affecting the organ microenvironment. Clin Exp Metastasis.

[67] Burger M, Glodek A, Hartmann T, Schmitt-Graff A, Silberstein LE, Fujii N, Kipps TJ, Burger JA (2003). Functional expression of CXCR4 (CD184) on small-cell lung cancer cells mediates migration, integrin activation, and adhesion to stromal cells. Oncogene.

[68] Taromi S, Kayser G, Catusse J, von Elverfeldt D, Reichardt W, Braun F, Weber WA, Zeiser R, Burger M (2016). CXCR4 antagonists suppress small cell lung cancer progression. Oncotarget.

[69] Huang S, Ernberg I, Kauffman S (2009). Cancer attractors: a systems view of tumors from a gene network dynamics and developmental perspective. Semin Cell Dev Biol.

[70] Jia D, Jolly MK, Kulkarni P, Levine H (2017). Phenotypic Plasticity and Cell Fate Decisions in Cancer: Insights from Dynamical Systems Theory. Cancers (Basel).

[71] Ao P, Galas D, Hood L, Zhu X (2008). Cancer as robust intrinsic state of endogenous molecular-cellular network shaped by evolution. Med Hypotheses.

[72] Li C, Wang J (2014). Quantifying the underlying landscape and paths of cancer. J R Soc Interface.

[73] Reck M, Heigener D, Reinmuth N (2016). Immunotherapy for small-cell lung cancer: emerging evidence. Future Oncol.

[74] Hogeweg P (2010). Multilevel Cellular Automata as a Tool for Studying Bioinformatic Processes.

[75] Macklin P, Edgerton M (2010). Multiscale modeling of Cancer - An Integrated Experimental and Mathematical Modeling Approach.

[76] Anderson A, Rejniak K, Gerlee P, Quaranta V (2007). Modelling of Cancer Growth, Evolution and Invasion: Bridging Scales and Models. Math Model Nat Phenom.

[77] Wu F (1982). The potts model. Reviews of Modern Physics.

[78] Steinberg M (1975). Adhesion-guided multicellular assembly: a commentary upon thepostulates, real and imagined, of the differential adhesion hypothesis, with special attention to computer simulations of cell sorting. Journal of Theoretical Biology.

[79] Mansury Y, Kimura M, Lobo J, Deisboeck T (2002). Emerging patterns in tumor systems: simulating the dynamics of multicellular clusters with an agent-based spatial agglomeration model. J Theor Biol.

[80] Anderson A (2005). A hybrid mathematical model of solid tumor invasion: the importance of cell adhesion. Math Med Biol.

[81] Ghaffarizadeh A, Friedman SH, Macklin P (2016). BioFVM: an efficient, parallelized diffusive transport solver for 3-D biological simulations. Bioinformatics.

[82] Gardner M (1970). Mathematical Games - The fantastic combinations of John Conway's new solitaire game “life”. Scientific American.

[83] Dormann S, Deutsch A (2002). Modeling of self-organized avascular tumor growth with a hybrid cellular automaton. In Silico Biol.

[84] Kansal AR, Torquato S, Harsh GI, Chiocca EA, Deisboeck TS (2000). Simulated brain tumor growth dynamics using a three-dimensional cellular automaton. J Theor Biol.

[85] Kimmel M, Axelrod D (1991). Unequal cell division, growth regulation and colony size of mammalian cells: a mathematical model and analysis of experimental data. J Theor Biol.

[86] Qi AS, Zheng X, Du CY, An BS (1993). A cellular automaton model of cancerous growth. J Theor Biol.

[87] Smolle J, Stettner H (1993). Computer simulation of tumour cell invasion by a stochastic growth model. J Theor Biol.

[88] Düchting W (1990). Tumor growth simulation. Comput Graph.

[89] Düchting W, Ulmer W, Ginsberg T (1996). Cancer: a challenge for control theory and computer modelling. Eur J Cancer.

[90] Enderling H, Chaplain MA (2014). Mathematical Modeling of Tumor Growth and Treatment. Current Pharmaceutical Design.

[91] Hahnfeldt P, Panigrahy D, Folkman J, Hlatky L (1999). Tumor development under angiogenic signaling: a dynamical theory of tumor growth, treatment response, and postvascular dormancy. Cancer Res.

[92] Gatenby R, Gawlinski E (1996). A reaction-diffusion model of cancer invasion. Cancer Res.

[93] Anderson A, Chaplain M, Newman E, Steele R, Thompson A (2000). Mathematical modelling of tumour invasion and metastasis. Computational Mathematical Methods Med.

[94] Anderson A, Chaplain M (1998). Continuous and Discrete Mathematical Models of Tumor-induced Angiogenesis. Bulletin of Mathematical. Biology.

[95] Itik M, Banks S (2010). Chaos in a three-dimensional Cancer Model. International Journal of Bifurcation and Chaos.

[96] Rietman E, Karp R, Tuszynski J (2011). Review and application of group theory to molecular systems biology. Theoretical Biology and Medical Modelling.

[97] Shahzad M (2016). Chaos Control in Three Dimensional Cancer Model by State Space Exact Linearization Based on Lie. Algebra Mathematics.

[98] Havlin S, Buldyrev SV, Goldberger AL, Mantegna RN, Ossadnik SM, Peng CK, Simons M, Stanley HE (1995). Fractals in Biology and Medicine. Chaos, Solitons and Fractals.

[99] Stojić T, Reljin I, Reljin B (2006). Adaptation of multifractal analysis of to segmentation of microcalcifications in digital mammograms. Physica A: Statistical Mechanics and its Applications.

[100] Reljin I, Reljin B (2002). Fractal geometry and multifractals in analyzing and processing medical data and images. Archive of Oncology.

[101] Falconer K (1990). Fractal Geometry - Mathematical Foundations and Applications.

[102] Levy-Vehel J, Berroir J Multifractals, texture, and image analysis.

[103] Hemsley A, Mukundan R Multifractal Measures for Tissue Image Classification and Retrieval.

[104] Lopes R, Betrouni N (2009). Fractal and Multifractal Analysis: A review. Medical Image Analysis.

[105] Mukundan R, Hemsley A (2010). Tissue Image Classification Using Multifractal Spectra. International Journal of Multimedia Data Engineering and Management.

[106] Theiler J (1990). Estimating Fractal Dimension. J Opt Soc Am A.

[107] Vasiljevic J, Pribic J, Kanjer K, Jonakowski W, Sopta J, Nikolic-Vukosavljevic D, Radulovic M (2015). Multifractal analysis of tumor microscopic images in the prediction of breast cancer chemotherapy response. Biomed Microdevices.

[108] Romanidou O, Imbimbo M, Mountzios G, Abidin A, Morgillo F, Califano R (2016). Therapies in the pipeline for small-cell lung cancer. Br Med Bull.

[109] Pietanza MC, Zimmerman S, Peters S, Curran WJ (2016). Seeking New Approaches to Patients With Small Cell Lung Cancer. Am Soc Clin Oncol Educ Book.

[110] Tan WL, Jain A, Takano A, Newell EW, Iyer NG, Lim WT, Tan EH, Zhai W, Hillmer AM, Tam WL, Tan DS (2016). Novel therapeutic targets on the horizon for lung cancer. Lancet Oncol.

[111] Roviello G, Zanotti L, Cappelletti MR, Gobbi A, Senti C, Bottini A, Generali D (2016). No Advantage in Survival With Targeted Therapies as Maintenance in Patients With Limited and Extensive-Stage Small Cell Lung Cancer: A Literature-Based Meta-Analysis of Randomized Trials. Clin Lung Cancer.

[112] Koinis F, Kotsakis A, Georgoulias V (2016). Small cell lung cancer (SCLC): no treatment advances in recent years. Transl Lung Cancer Res.

[113] Frost JJ, Pienta KJ, Coffey DS (2018). Symmetry and symmetry breaking in cancer: a foundational approach to the cancer problem. Oncotarget.

[114] Benzekry S, Gandolfi A, Hahnfeldt P (2014). Global dormancy of metastases due to systemic inhibition of angiogenesis. PLoS One.

