# Supplementary material for: Modeling small cell lung cancer (SCLC) biology through deterministic and stochastic mathematical models
Source: Oncotarget. 2018 May 25;9(40):26226–42. doi: 10.18632/oncotarget.25360 (PMC5995226; doi:10.18632/oncotarget.25360)
Supplement: Supplementary file 1 [file oncotarget-09-26226-s001.pdf]

Modeling small cell lung cancer (SCLC) biology through deterministic and stochastic mathematical models

SUPPLEMENTARY MATERIALS

Supplementary Video 1: Most common metastasis sites of SCLC. Simulation of early time points and the endpoint (from top left to bottom right) of tumor cells (purple) metastasizing to different organs (colored as indicated). See Supplementary_Video_1

Supplementary Video 2: The cellular automaton model of SCLC growth and necrosis. Growth of tumor (scale on left side) and oxygen levels within the tumor (color scale on right side) at different time points. See Supplementary_Video_2

Supplementary Video 3: The cellular automaton model of SCLC growth and necrosis. Growth of tumor showing the extent of the necrosis at different times (red = live cells, green = apoptotic cells, and blue = necrotic cells). The necrosis of the tumor initiates at > 180 hours. See Supplementary_Video_3

Supplementary Video 4: Simulation of tumor heterogeneity and tumor burden in SCLC. Tumor heterogeneity and cellular automata at different time points. Tumor burden at corresponding time points, indicating the growth of wild-type cells (red) and cells containing mutations (blue). See Supplementary_Video_4
